# Similarity of Seroma Rate at the Medial Thigh following Free Flap Harvesting or Medial Thigh Lift: A Systematic Review and Meta-analysis

**DOI:** 10.1016/j.jpra.2024.03.013

**Published:** 2024-04-05

**Authors:** K.K. Kilian, A.C. Panayi, D.Y. Matar, C. Hamwi, A.K. Bigdeli, U. Kneser, F.H. Vollbach

**Affiliations:** a Department of Hand, Plastic and Reconstructive Surgery, Microsurgery, Burn Center, BG Center Ludwigshafen, Ludwig-Guttmann-Strasse 13, 67071 Ludwigshafen, Germany; b Department of Hand and Plastic Surgery, University of Heidelberg, Im Neuenheimer Feld 672, 69120 Heidelberg, Germany; c Department of Surgery, Division of Plastic Surgery, Brigham and Women's Hospital, Harvard Medical School, 75 Francis St, Boston, MA 02115, USA; d Johns Hopkins University School of Medicine, Baltimore, MD, USA; e Department of Data Science, Saint Louis University, St. Louis, MO, USA; f Division of Hand, Plastic and Aesthetic Surgery, Ludwig-Maximilians-University (LMU), Munich, Germany

**Keywords:** Autologous breast reconstruction, Seroma, Transverse upper gracilis flap, Profunda artery perforator flap, Medial thigh lift

## Abstract

Despite the growing use of autologous breast reconstruction with medial thigh-based free flaps, such as transverse upper gracilis (TMG) or profunda artery perforator (PAP) flaps, these procedures are infrequently performed on patients with obesity. This systematic review and meta-analysis aimed to compare the frequency of seroma occurrence, a common complication after medial thigh flap surgery. Comparison was performed between TMG and PAP flaps, as well as medial thigh lifts (MTL), a procedure with a similar operative technique but which is typically offered to patients with a higher body mass index (BMI).

Following the Preferred Reporting Items for Systematic Reviews and Meta-analyses guidelines, we analyzed EMBASE, PUBMED, and MEDLINE data (English/German). The primary outcomes assessed were occurrence of seroma, as well as hematoma and wound dehiscence. Subgroup analyses explored age, BMI, and various surgical factors.

This meta-analysis incorporated 28 studies, totaling 1096 patients. MTL patients had significantly higher BMIs, whereas seroma rates were similar among TMG, PAP, and MTL patients. The incidence of hematoma and wound dehiscence was also similar across the groups. In the metaregression analysis, factors such as age and BMI showed no significant correlation with seroma occurrence in all groups.

This systematic review and meta-analysis identified comparable rates of seroma formation after TMG flap, PAP flap, and MTL procedures. Considering that this phenomenon occurred despite the elevated BMI of the MTL group, we propose that patients with higher BMI need not be excluded as candidates for autologous medial thigh-based breast reconstruction. Hence, these procedures should not be limited to small- to medium-sized breasts. Large-scale prospective studies are imperative to validate these conclusions and reveal the underlying factors contributing to seroma formation.

## Introduction

Seroma formation, defined as serous fluid collection under the skin following surgery, is one of the most common surgical sequelae, occurring not only after free flap harvest^1^ but also after aesthetic procedures of the upper medial thigh.^2^ The seroma occurrence rate after free flap harvest from the medial thigh or thigh plasty ranges between 0% and 29% in the literature.^3^, ^4^, ^5^, ^6^, ^7^, ^8^, ^9^, ^10^, ^11^, ^12^, ^13^, ^14^, ^15^, ^16^, ^17^, ^18^, ^19^, ^20^, ^21^, ^22^, ^23^, ^24^, ^25^, ^26^, ^27^, ^28^, ^29^, ^30^ Patients with seroma experience discomfort and are at an elevated risk of developing wound complications such as infection, wound dehiscence, and necrosis. Additionally, seroma can contribute to an extended recovery period with prolonged aftercare treatment and, hence, higher medical expenses.^31^, ^32^, ^33^ Therefore, its significance in postoperative care is considerable.

Free flaps harvested from the upper medial thigh, including transverse upper gracilis (TMG) and profunda artery perforator (PAP) flaps, play a vital role, particularly in autologous reconstructive breast surgery. The demand for autologous breast reconstruction utilizing the thigh as a donor site is on the rise, driven by a focus on addressing the individualized requirements of each patient^34^ and the avoidance of common complications associated with breast implants, such as extrusion, capsular contracture, implant malposition, or rupture.^35^ As a result, breast reconstruction using medial thigh flaps has become a pivotal treatment choice, necessitating a comprehensive understanding of surgical repercussions. The complications associated with medial thigh flap harvest are primarily investigated in patients with lower body mass index (BMI), as the TMG flap is exclusively considered the leading option for free flap reconstruction in slim and healthy-weight individuals who may lack sufficient abdominal donor tissue.^36^

In contrast, medial thigh lift (MTL) procedures involve the removal of excess skin and fat from the inner portion of the thigh,^2^ primarily in patients with significant weight loss. Due to the growing prevalence of obesity among surgical patients, with projections estimating that in the year 2030 approximately 80% of surgical patients will have an above-normal BMI,^37^^,^^38^ there is a corresponding increase in the demand for bariatric and postbariatric surgeries.^39^ The incision line and tissue removal involved in upper horizontal thigh lift procedures exhibit similarities to harvesting a medial thigh flap, albeit with the latter necessitating a deeper tissue advancement and, in the case of TMG flaps, requiring muscle resection.

With this comprehensive review and meta-analysis, this study aimed to compare the incidence of seroma formation in MTL procedures and included upper thigh lifts to encompass a wider spectrum of BMI in evaluating seromas in patients with different BMIs. Due to the increasing prevalence of obesity as a prevailing concern in industrialized nations,^40^ MTL procedures, as a category sharing similar operative methods but performed in patients with higher BMI, bear significance.

## Patients and Methods

### Data Source

The PubMed, EMBASE, Web of Science, and Google Scholar databases were searched from inception to March 31, 2023. The following search terms were used: seroma, thigh, thigh lift, transverse musculocutaneous gracilis flap, PAP flap, and transverse upper gracilis. The search format was tailored to the syntax of each database, and the meta-analysis was conducted following the Preferred Reporting Items for Systematic Reviews and Meta-Analyses (PRISMA) statement.^41^ The meta-analysis was registered a priori on Prospero (registration no. CRD42023405323).

### Study Identification and Selection

The studies were identified independently by two authors (FV and KK) following a three-stage process. First, all eligible articles were screened based on the title and abstract. In a second step, the abstracts of all possibly eligible articles were assessed based on inclusion and exclusion criteria. In the third and final stage, the manuscripts of all remaining studies were analyzed, and eligibility was carefully screened against inclusion or exclusion criteria. During the screening process, the reference lists of each included study and any systematic reviews or meta-analyses that were reviewed were manually examined to identify pertinent literature.

### Inclusion and Exclusion Criteria

The included studies had to fulfill the following criteria: (a) reporting on patients undergoing free flap harvesting from the medial thigh (TMG flap, PAP flap, or MTL procedures), including (b) a horizontal incision of the upper medial thigh; (c) reporting on seroma formation; (d) published in English or German; and (f) having a prospective or retrospective study design, including cohort studies, randomized control trials, case series, case-control studies, and clinical trials. Studies were screened for first author's name, publication year, study design, number of patients, comorbidities (e.g., diabetes), and primary and secondary outcomes.

Studies on surgical interventions of the medial thigh involving solely a vertical incision, as well as non-English or non-German language studies, and any basic science, animal, or cadaver studies were excluded.

### Data Extraction

Data were extracted by two independent investigators (KK and FV) using Microsoft Excel^Ⓡ^ 2020 (Microsoft, Redmond, WA, USA) and included the first author's name, year of publication, country of the treating institution, type of study (prospective or retrospective), level of evidence, surgical method, total number of patients, number of flaps, patient demographics (sex, age, BMI, and comorbidities), and surgical characteristics (surgical method, length of operation, and flap size and weight). Outcomes details were compiled, including complications (seroma, hematoma, and dehiscence) and length of hospital stay (LOHS).

### Outcomes

The primary outcome was a seroma at the upper medial thigh. Secondary outcomes included further complications of the thigh (hematoma and wound dehiscence) and additional details (age, BMI, flap size, flap weight, operation time [OT], and LOHS).

### Quality Assessment

The Grading of Recommendations, Assessment, Development and Evaluations (GRADE) tool^42^ was employed to assess the quality of the included studies. The assessment was performed independently by two authors (DYM and CH). Any inconsistencies in the quality assessment were resolved by the corresponding author (FV).

### Statistical Analysis

Data were collected in Microsoft Excel^Ⓡ^ 2020. A meta-analysis of proportions was performed in Open Meta (Analyst) (version 10.12 for Mac OS) following the Cochrane Collaboration and the Quality of Reporting of Meta-analyses statement.^43^ Heterogeneity was evaluated with the I^2^ statistic. The random-effects model with the DerSimonian and Laird variance estimator was used to calculate pooled proportions (%) or weighted mean and standard deviation (SD) with a 95% confidence interval (95% CI).

The estimates for the pooled proportions were transformed using the Freeman-Tukey double-arcsine transformation to achieve normal distribution.^44^

According to the Freeman-Tukey double-arcsine transformation model, the estimate of the pooled outcome prevalence (pi) is calculated as $p_{i}=\frac{e_{i}}{n_{i}}$, where ei is the number of events (i.e. of seroma, hematoma, and dehiscence) and ni is the total sample size of the included studies.^45^ The weighted pooled estimates are first calculated, and a back-transformation is performed on these estimates to stabilize any within-study variance through binomial distribution. The following transformation equation is used:

$$y_{i}\;=\;g\left(p_{i}\right)\;=\;arsin\sqrt{\frac{ei}{ni+1}}\;+\;arsin\sqrt{\frac{ei\;+\;1}{n\;+\;1}}$$

where variance (vi) is equal to $\frac{1}{n_{i}+0.5}$ . When SDs were not provided in the study data, they were indirectly calculated from the interquartile range or standard error or imputed from similar studies in terms of composition and size of the cohort.

The significance of the difference between the estimates was determined using an unpaired t-test, as described previously.^46^ Metaregressions were performed using Open Meta (Analyst) (version 10.12 for Mac OS) to investigate the correlation between seroma occurrence and median age, BMI, and resection weight (including bilateral pannus weight and flap weight) in grams. Significance was established when p < 0.05. Data were visualized in GraphPad Prism 9 and Adobe Illustrator (version 2022).

## Results

### Studies Included in the Meta-analysis

**Figure 1:**
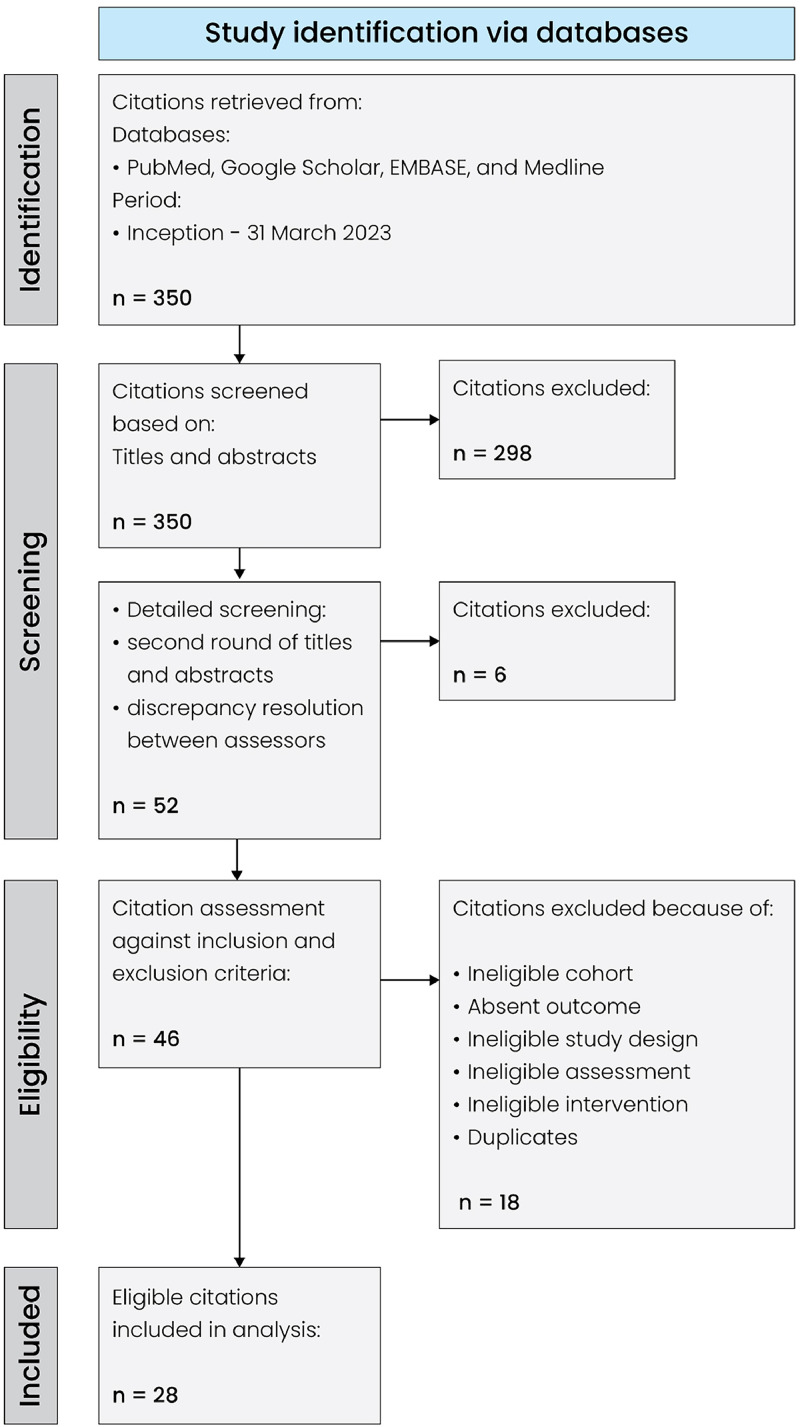
PRISMA flow diagram of the study identification process.

During the initial database search, a total of 350 studies were identified (Figure 1). Following title and abstract evaluation, 298 studies were excluded. After a detailed assessment, 24 studies were excluded due to the lack of original or relevant data and ineligible cohorts. Finally, a total of 28 studies comprising 1,096 patients were included in the meta-analysis (Table 1). Seventeen studies presented data on TMG flaps,^10^, ^11^, ^12^, ^13^, ^14^, ^15^, ^16^, ^17^, ^18^, ^19^, ^20^, ^21^, ^22^, ^23^, ^24^, ^25^, ^26^ five studies on PAP flaps,^17^^,^^27^, ^28^, ^29^, ^30^ and six studies on MTL.^3^, ^4^, ^5^, ^6^, ^7^, ^8^, ^9^ Nearly all studies (96%), except for the study by Candiani et al. (1995),^7^ were published after 2005. Six (21%) studies were published after 2020.

**Table 1:** Characteristics of all included studies and cohorts.

Study	Year	Country	Type of study	Reconstructive technique	No. of patients
Atzeni et al.	2022	Italy	R	PAP flap	86
Augustin et al.	2023	Austria	R	PAP flap	18
Bertheuil et al.	2014	France	R	MTL	21
Bertheuil et al.	2013	France	R	MTL	53
Bodin et al.	2015	France	R	TMG flap	36
Bracaglia et al.	2015	Italy	R	MTL	16
Bruschi et al.	2008	Italy	R	MTL	35
Buntic et al.	2010	USA	R	TMG flap	20
Candiani et al.	1995	Italy	R	MTL	18
Cho et al.	2019	USA	R	PAP flap	69
Ciudad et al.	2015	Italy	R	PAP flap	9
Craggs et al.	2014	Belgium	R	TMG flap	36
Di Pietro et al.	2020	Italy	R	MTL	26
Eom et al.	2011	Korea	R	TMG flap	40
Fansa et al.	2008	Germany	R	TMG flap	20
Fattah et al.	2008	UK	R	TMG flap	12
Gusenoff et al.	2014	USA	R	MTL	38
Hunter et al.	2015	UK	R	TMG/PAP flap	54/22
Locke et al.	2012	Canada	R	TMG flap	8
Merchant et al.	2022	Switzerland	R	TMG flap	20
Nickl et al.	2017	Austria	R	TMG flap	34
Saint-Cyr et al.	2012	France	R	TMG flap	13
Saour et al.	2017	France	R	TMG flap	12
Schoeller et al.	2007	Austria	R	TMG flap	111
Siegwart et al.	2021	Germany	R	TMG flap	99
Vollbach et al.	2014	Germany	R	TMG flap	98
Weitgasser et al.	2020	Germany	R	TMG flap	43
Werdin et al.	2015	Germany	R	TMG flap	29

Abbreviations: R, Retrospective; PAP, profunda artery perforator; MTL, medial thigh lift; TMG, transverse musculocutaneous gracilis.

Some geographical distribution was seen, with 23 (82%) studies published in Europe, 4 (14%) studies published in North America, and 1 (3.6%) study in Asia. All studies were designed retrospectively.

The total number of free flaps from the medial thigh was 1,244 [949 (76.3%) TMG flaps and 295 (23%) PAP flaps, respectively]. A total of 207 MTL procedures were reported in the included studies. The pooled mean age of the TMG cohort was 48.6 ± 1.3 years, with a mean BMI of 22.3 ± 0.3 kg/m^2^. The mean age of the PAP cohort was 50.3 ± 1.8 years, with a mean BMI of 23.1 ± 1.2 kg/m^2^. Finally, the mean age of the MTL cohort was 47.0 ± 0.9 years, with a mean BMI of 35.2 ± 2.7 kg/m^2^. No significant differences between the cohorts were noted in terms of age (p > 0.1), although the MTL group significantly differed from the TMG group (p = 0.0001) and the PAP group (p = 0.0003) in terms of BMI. The TMG and PAP groups had similar BMI (p = 0.26).

Collectively, although there were comparable ages among the cohorts (p > 0.1), the MTL group had a notably elevated mean BMI, surpassing the BMIs of the TMG and PAP groups by >10 kg/m^2^.

### Quality Assessment

**Figure 2:**
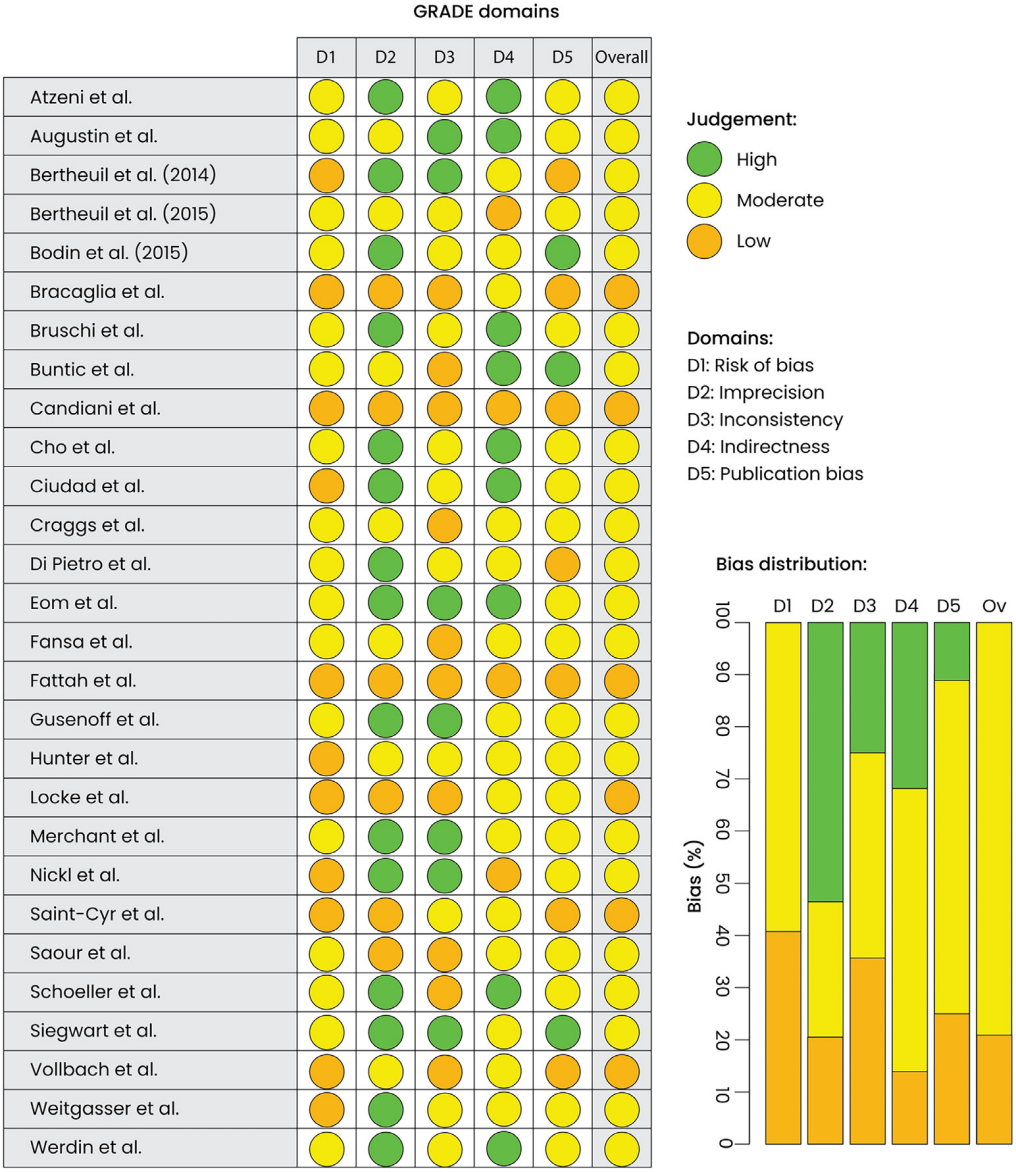
Quality assessment of the studies included in the pairwise meta-analysis study based on the GRADE tool. Left panel: “traffic light” visualization of the domain bias of each individual paper. Right panel: weighted bar plots of the distribution of bias within each domain.

Quality assessment of the included articles using the GRADE tool showed that 78% were moderate quality and 21% were low quality. A “traffic light” visualization of the domain bias of each individual paper, as well as weighted bar plots of the distribution of bias within each domain, is shown in Figure 2.

### Primary Outcome

**Figure 3:**
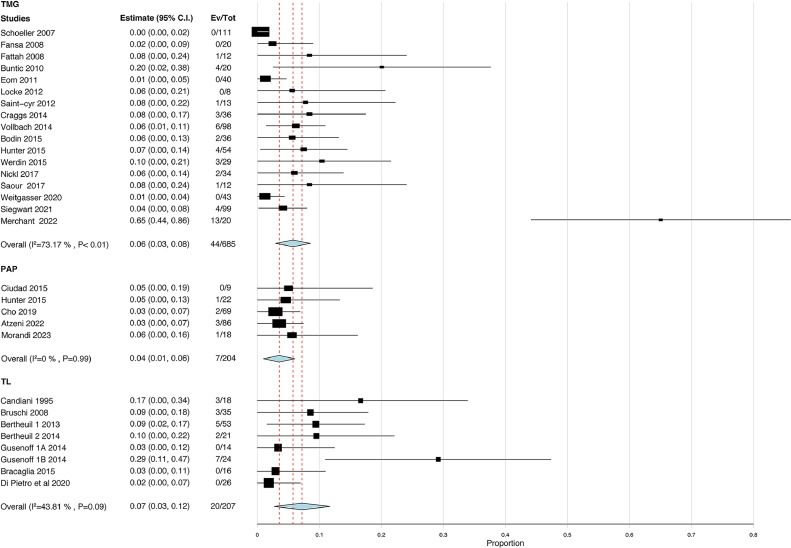
Forest plots of seroma occurrence. Heterogeneity was significantly high in the TMG group. The pooled rate of seroma occurrence was lowest in the PAP group and highest in the MTL group. Abbreviations: TMG, transverse musculocutaneous gracilis; PAP, profunda artery perforator; MTL, medial thigh lift.

Data on seroma occurrence were provided in 17 studies focusing on TMG flaps (n = 685), 5 studies on PAP flaps (n = 204), and 6 studies on MTL (n = 207; Figure 3). The pooled rate of seroma occurrence was lower in the PAP group (3.4%; estimate = 0.04; 95% CI = 0.01-0.06) than in the TMG group (6.4%; estimate = 0.06; 95% CI = 0.03-0.08; p = 0.30) and MTL group (9.7%; estimate = 0.07; 95% CI = 0.03-0.12; p = 0.18), although none of these were significant (Table 2). Furthermore, the seroma rates were not statistically different between the TMG and MTL groups (p = 0.64). In aggregate, there was no difference in seroma occurrence in all groups.

**Table 2:** Primary and secondary outcomes as calculated from pooled proportions MA.

Primary outcome	Studies	Patients	Estimate (95% CI)	I^2^ %	p-value
Seroma					
TMG	17	685	0.06 (0.03, 0.08)	73.17	<0.01
PAP	5	204	0.04 (0.01, 0.06)	0.00	0.99
MTL	8	207	0.07 (0.03, 0.12)	43.81	0.09
Secondary outcome					
Hematoma					
TMG	6	305	0.01 (0.00, 0.03)	0.00	0.84
PAP	3	173	0.03 (0.00, 0.06)	22.44	0.28
MTL	6	133	0.04 (0.01, 0.07)	0.00	0.48
Dehiscence					
TMG	11	523	0.13 (0.08, 0.18)	62.30	<0.01
PAP	5	204	0.29 (0.03, 0.54)	97.62	<0.01
MTL	6	138	0.20 (0.08, 0.33)	84.19	<0.01

Results presented for the three groups, TMG, PAP and MTL. P values represent the significance of the heterogeneity.

CI, confidence interval; I^2^, heterogeneity.

### Secondary Outcomes

**Figure 4:**
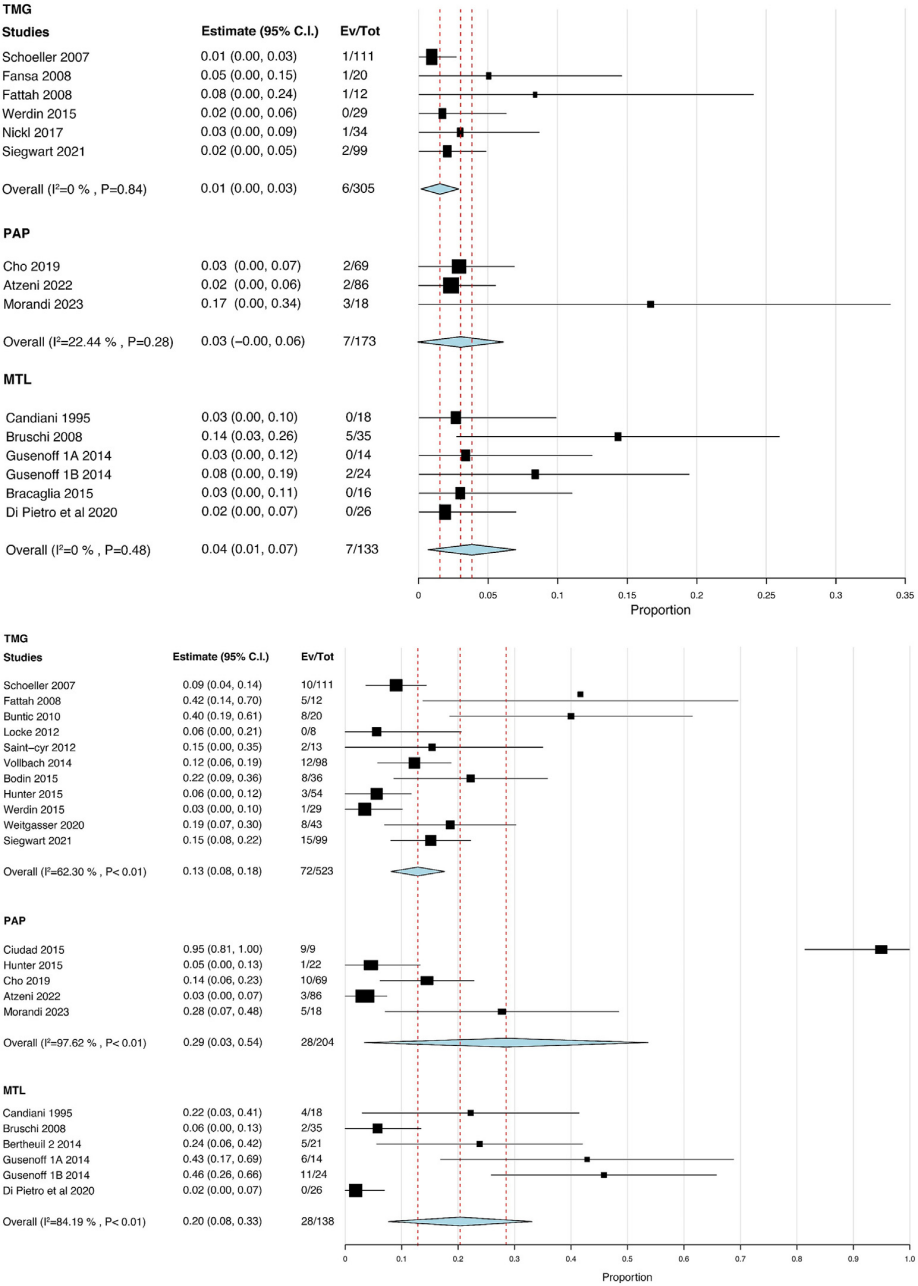
Forest plots of the secondary outcomes. Hematoma occurrence (a): Heterogeneity was low in all groups. The pooled rate of hematoma occurrence was lowest in the TMG group and highest in the MTL group. Dehiscence occurrence (b): Heterogeneity was significantly high in all groups (p < 0.01). The pooled rate of dehiscence occurrence was lowest in the TMG group and highest in the PAP group. Abbreviations: TMG, transverse musculocutaneous gracilis; MTL, medial thigh lift; PAP, profunda artery perforator.

Data on hematoma occurrence were provided in 6 (21%) studies focusing on TMG flaps (n = 305), 3 (10%) studies on PAP flaps (n = 173), and 6 (21%) studies on MTL (n = 133; Figure 4a). The pooled rate was lower in the TMG group (2.0%; estimate = 0.01; 95% CI = 0.00-0.03) than in the PAP group (4.0%; estimate = 0.03; 95% CI = 0.00-0.06; p = 0.73) and MTL group (5.3%; estimate = 0.04; 95% CI = 0.01-0.07; p = 0.14). The hematoma rates were also similar between the PAP and MTL groups (p = 0.32). Not all differences were significant (Table 2).

Data on wound dehiscence occurrence were provided in 11 (39%) studies focusing on TMG flaps (n = 523), 5 (17%) studies on PAP flaps (n = 204), and 6 (21%) studies on MTL (n = 138; Figure 4b). The pooled rate was lower in the TMG group (13%; estimate = 0.13; 95% CI = 0.08-0.18) than in the PAP group (13%; estimate = 0.29; 95% CI = 0.03-0.54; p = 0.07) and MTL group (20%; estimate = 0.20; 95% CI = 0.08-0.33; p = 0.64). The wound dehiscence rates were also similar between the PAP and MTL groups (p = 0.59), with no significant difference noted (Table 2).

Taken as a whole, there was no evident discrepancy in hematoma and wound dehiscence between free flaps from the medial thigh and upper vertical thigh lift procedures. However, a noticeable trend suggested that the TMG flap has lower rates of both complications.

### Metaregression of Seroma Occurrence

Metaregression in the TMG group identified a positive correlation between median age and seroma occurrence (coefficient = 0.004; 95% CI = 0-0.008; p = 0.07). A negative correlation was seen between BMI and seroma occurrence (coefficient = -0.007; 95% CI = −0.007 to 0.06; p = 0.84). Flap size (coefficient = 0; 95% CI = 0-0.001; p = 0.82), flap weight (coefficient = 0; 95% CI = 0-0; p = 0.77), OT (coefficient = 0; 95% CI = −0.001 to 0; p = 0.14), and LOHS (coefficient = 0; 95% CI = −0.05 to 0.05; p = 0.99) were linearly related to seroma occurrence. None of these correlations were significant.

Metaregression in the PAP group identified a positive correlation between median age and seroma occurrence (coefficient = 0.001; 95% CI = −0.002 to 0.004; p = 0.66). A negative correlation was seen between BMI and seroma occurrence (coefficient = −0.004; 95% CI = −0.03 to 0.02; p = 0.79). Flap weight (coefficient = 0; 95% CI = −0.001 to 0.001; p = 0.79) was linearly related to seroma occurrence. None of these correlations were significant.

**Figure 5:**
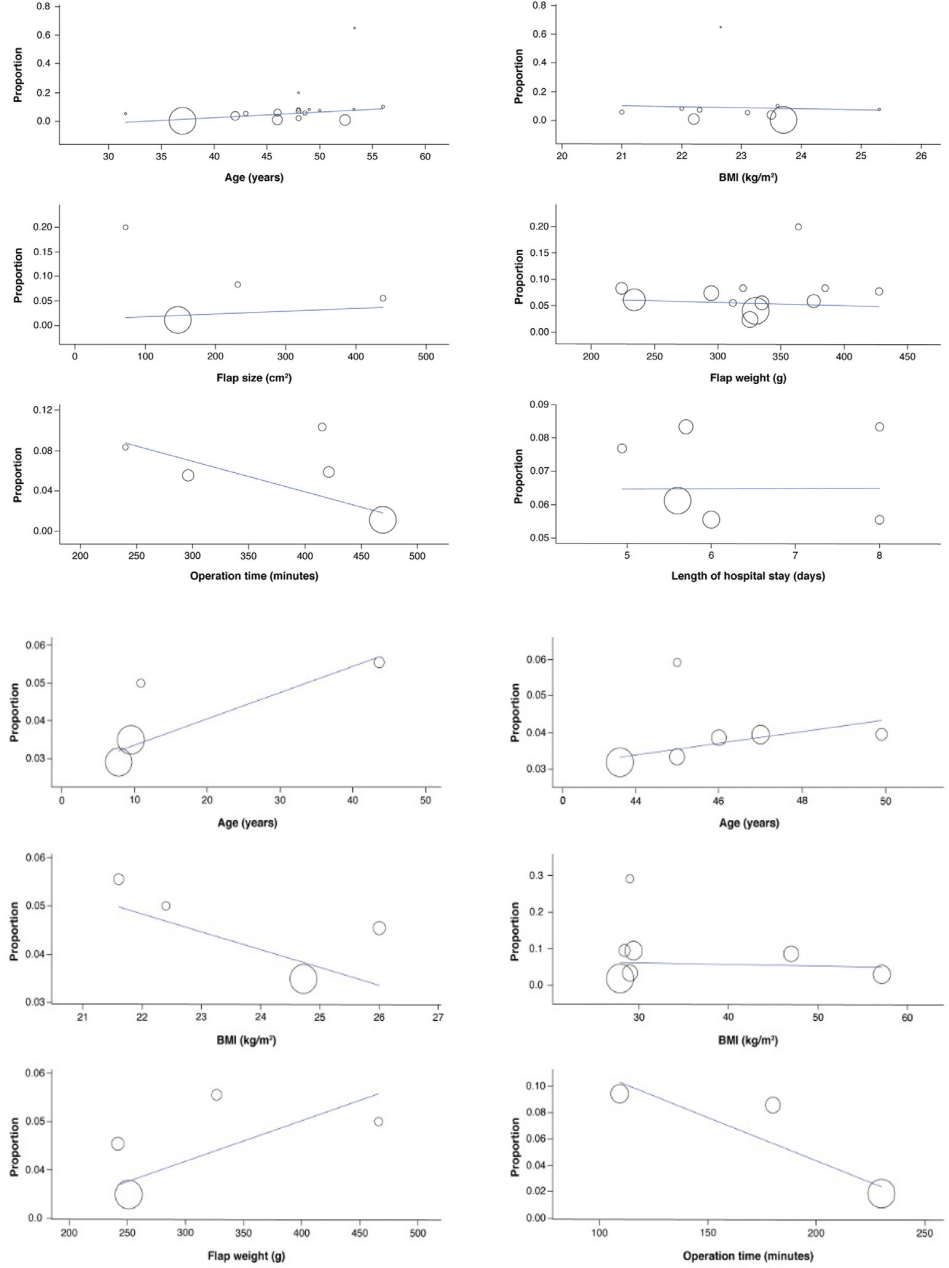
Meta-regression analysis of seroma occurrence: (a) in the TMG studies: Correlation with age, BMI, flap size, flap weight, operation time and length of hospital stay was assessed; (b) in the PAP studies: Correlation with age, BMI, and flap weight was assessed; (c) in the MTL studies: Correlation with age, BMI, and operation time was assessed. Abbreviations: TMG, transverse musculocutaneous gracilis; BMI, body mass index; PAP, profunda artery perforator; MTL, medial thigh lift.

Metaregression in the MTL group identified a positive correlation between median age and seroma occurrence (coefficient = 0.02; 95% CI = −0.002 to 0.03; p = 0.09). A negative correlation was seen between OT and seroma occurrence (coefficient = -0.001; 95% CI = −0.001 to 0; p = 0.10). BMI (coefficient = 0; 95% CI = −0.004 to 0.003; p = 0.78) was linearly related to seroma occurrence. Again, none of these correlations were significant. The metaregression graphs are shown in Figure 5a to c.

Metaregression findings showed no significant correlation between age and BMI in MTL or after medial thigh flap procedures with respect to seroma occurrence.

## Discussion

The high incidence of seroma occurrence after surgery at the medial thigh can be partly explained by an extensive lymphatic network in the medial thigh. The subcutaneous ventromedial bundle, which is part of the superficial system of the great saphenous vein, is particularly susceptible to damage during surgical interventions in the medial thigh area, and the disruption of lymphatic vessels can contribute to seroma formation.^47^

Medial thigh flaps, such as TMG and PAP flaps, have gained popularity in reconstructive surgery due to their reliable vascular anatomy and straightforward tissue harvesting.^48^ However, it is imperative to consider the occurrence rate of donor-site complications, such as seroma formation, which is one of the most common complications associated with these flaps^1^ and can significantly reduce the quality of life and considerably prolong recovery time, thus delaying urgent adjuvant therapy and potentially hampering oncological outcomes.^49^

Initially, obesity was identified as a potential risk factor for complications after abdominal-based free flap breast reconstruction.^50^ Obesity is still recognized as a general risk factor for complications in microsurgical breast reconstruction.^36^ A previous meta-analysis that examined the impact of obesity on breast reconstruction outcomes highlighted a connection between BMI > 30 and an increased occurrence of seroma.^51^ Nevertheless, the status of obesity as a common risk factor is still a subject of controversial discussion.^52^ Recent findings from free TMG flap breast reconstructions suggested that being overweight does not significantly increase the risk of postoperative complications.^36^ Considering the scarcity of data on patients with obesity undergoing medial thigh free flap procedures, this study incorporated individuals who primarily underwent upper horizontal thigh lifts, sharing comparable operative techniques and encompassing a range of higher BMIs. After thighplasty, seroma formation is still a significant donor-site complication.^2^

In this systematic review and meta-analysis, the MTL group showed a notably elevated mean BMI, surpassing the BMI of the TMG and PAP groups. Although this difference was given, the pooled rates of seroma occurrence did not significantly differ among the TMG, PAP, and MTL groups. Therefore, a higher BMI does not correlate with increased seroma formation. Moreover, the metaregression analysis demonstrated decreased seroma rates with increasing BMI. Recognizing the increasing prevalence of obesity, it becomes crucial to acknowledge the importance of free flaps based on the medial thigh as a viable treatment option for patients with obesity, particularly in the context of the ongoing need for autologous breast reconstruction.^34^

Zieliński et al. noted that patients >60 years old suffered from higher total seroma volumes and longer treatment times for seroma formation compared to younger patients.^53^ However, the metaregression analyses investigated impact factors such as age, BMI, flap size and weight, OT, and LOHS and showed no significant correlation with seroma occurrence in the TMG, PAP, or MTL groups.

For the secondary outcomes, there were no statistically significant differences in the pooled data on hematoma occurrence among the groups. Interestingly, although the TMG and PAP flap procedures involve muscle dissection,^54^^,^^55^ low heterogeneity was observed across all groups. The TMG flap also entails muscle harvest,^54^ potentially increasing the risk of hematoma formation. However, specific data regarding the muscle portion were missing, which limited further analysis in this regard. The pooled rates for hematoma formation were lower among TMG flaps than among PAP flaps. Similarly, data on dehiscence occurrence were not significantly different between the groups, albeit with high heterogeneity. Taken together, no specific procedure is associated with a higher frequency of complications, with all procedures relatively safe in terms of hematoma and wound dehiscence occurrence.

The study has several strengths, including a comprehensive search of multiple databases, adherence to PRISMA guidelines, and a registered protocol on Prospero. The use of sensitivity and metaregression analyses added to the robustness of the findings.

However, there are also limitations to consider. The inclusion of only English and German language studies may have introduced language bias. The retrospective nature of the included studies and the heterogeneity in surgical techniques and patient populations across studies may have limited the generalizability of the results. Overall, this systematic review and meta-analysis provided insights into the frequency of seroma formation after free flap harvesting from the medial thigh and MTL procedures. However, the included studies were predominantly retrospective, which may have introduced bias and limitations. Quality analysis of the studies identified that all were either low or moderate quality, which weakened the strength of this review. Furthermore, certain relevant factors, such as muscle incorporation in the flap, duration of drainage, use of negative pressure wound therapy (NPWT), discharge location, including details on the destination (e.g., home or specialized facilities), and perioperative serum albumin levels, were not reported in the included literature. For example, Athanassiou et al.^56^ highlighted that albumin polymer instillation reduces seroma during breast cancer surgery. Furthermore, NPWT studies identified reduced seroma rates in incisional wounds after hip surgery.^57^ Substantial heterogeneity and the overall low quality of the studies might account for the lack of statistical significance. Therefore, the predisposition of older patients or patients with obesity to seroma formation cannot be completely ruled out. High-quality prospective studies are needed to validate these findings and explore potential prognostic indicators of seroma formation by involving patients with a high BMI after medial thigh flap harvest.

## Conclusion

In conclusion, this systematic review and meta-analysis suggests that the frequency of seroma formation after TMG flaps, PAP flaps, and MTL procedures is comparable. Given that patients undergoing MTL have higher BMIs, the lack of difference in seroma occurrence may be a promising sign that higher BMI, and thus larger-sized breasts, is not a contraindication for autologous thigh-based breast reconstruction with the TMG or PAP flap, at least in terms of occurrence of one of the most common sequelae. Further research is warranted to better understand the underlying factors associated with seroma formation and guide clinical decision-making in managing patients undergoing these procedures.
